# At similar weight loss, dietary composition determines the degree of glycemic improvement in diet-induced obese C57BL/6 mice

**DOI:** 10.1371/journal.pone.0200779

**Published:** 2018-07-23

**Authors:** Roman Vangoitsenhoven, Miranda van der Ende, Katrien Corbeels, João Paulo Monteiro Carvalho Mori Cunha, Matthias Lannoo, Pierre Bedossa, Schalk van der Merwe, Ann Mertens, Ina Gesquiere, Ann Meulemans, Christophe Matthys, Chantal Mathieu, Lut Overbergh, Bart Van der Schueren

**Affiliations:** 1 Department of Chronic Diseases, Metabolism and Ageing, KU Leuven, Leuven, Belgium; 2 Department of Endocrinology, University Hospitals Leuven, Leuven, Belgium; 3 Department of Abdominal Surgery, University Hospitals Leuven, Leuven, Belgium; 4 Département d’Anatomie Pathologique, INSERM U773, Université Paris Diderot, Paris, France; 5 Laboratory of Hepatology, KU Leuven, Leuven, Belgium; 6 Department of Gastroenterology and Hepatology, University Hospitals Leuven, Leuven, Belgium; Universidade do Estado do Rio de Janeiro, BRAZIL

## Abstract

**Background:**

Achieving weight loss is the cornerstone of the treatment of the metabolic consequences of obesity, in particular of glucose intolerance.

**Objective:**

To determine whether improvement in glucose control depends on dietary macronutrient composition of the diet at identical weight loss.

**Materials and methods:**

Twenty-two weeks old diet-induced obese C57BL/6 mice lost weight through caloric restriction on normal chow (R-NC) or high fat diet (R-HF). Control mice were fed normal chow (LEAN) or high fat diet (OBESE) ad libitum. Body weight and composition were assessed after 8 weeks of dietary intervention. Glucose homeostasis was evaluated by intraperitoneal glucose tolerance tests (IPGTT). Epididymal white adipose (eWAT) and hepatic tissues were analyzed by immunohistochemistry and RT-qPCR.

**Results:**

By 30 weeks of age, the body weight of the mice on R-NC (31.6±1.7g, mean±SEM) and R-HF (32.3±0.9g) was similar to LEAN mice (31.9±1.4g), while OBESE mice weighed 51.7±2.4g. Glucose tolerance in R-NC was better than in LEAN mice (69% AUC IPGTT, P 0.0168) whereas R-HF mice remained significantly less glucose tolerant (125% AUC IPGTT, P 0.0279 vs LEAN), despite identical weight loss. The eWAT pads and adipocyte size were similar in LEAN and R-NC mice, while the eWAT pad size of R-HF was 180% of R-NC (P < 0.0001) and the average adipocyte size of R-HF mice was 134% of R-NC fed mice (P 0.0285). No LEAN or R-NC mice had hepatic steatosis, in contrast to 28.6% of R-HF mice. Compared to OBESE mice, inflammatory markers were lower in eWAT and liver tissue of R-NC, but not in R-HF mice. Measures of visceral adiposity correlated well with glucose tolerance parameters.

**Conclusions:**

In mice, caloric restriction on a normal chow diet improved glucose tolerance significantly more when identical weight loss was achieved on a high fat diet.

## Introduction

Despite the increase of the prevalence of obesity worldwide, a satisfactory non-invasive treatment is still awaited [1,2]. Caloric restriction remains the cornerstone of the treatment, since the excess weight is caused by a cumulative positive energy balance [3]. A multitude of dietary approaches to achieve caloric restriction and subsequent weight loss have been proposed [4] and several studies have compared diets in terms of efficacy, but so far it’s hard to draw firm conclusions on which strategy yields the best metabolic results [5–7]. Moreover, high dropout rates and poor adherence to the assigned diets have hampered interpretation of such studies in patients [5,8]. Moreover, some dietary interventions have even been criticized for worsening the metabolic consequences caused by obesity, increasing patients’ cardiovascular risk despite achieving weight loss [9,10]. The latter contrasts with the main goal to treat obesity, which is to improve or even reverse obesity related complications, in particular glucose intolerance, dyslipidemia and non alcoholic fatty liver disease [11]. These metabolic complications arise from ectopic fat storage which activates cellular stress and inflammatory responses, leading to insulin resistance, glucose intolerance and ultimately type 2 diabetes [12–14].

Our aim was to assess whether the composition of diets that achieve identical weight loss matters in terms of the metabolic improvement observed. Therefore, we compared the body composition and glucose homeostasis of lean mice to diet-induced obese mice that had lost all excess body weight (BW) through caloric restriction on either normal chow or a high fat diet.

## Materials and methods

### Animals and interventions

Eight weeks old male C57BL/6 mice (Charles River, Brussels, Belgium) were group housed in a conventional animalium (12h light/dark cycle at 22°C) and fed a high fat (HF) diet (60% kcal lard fat, D12492, Research Diets Inc., New Brunswick, NJ) for 14 weeks to achieve diet-induced obesity at 22 weeks of age. Subsequently, these mice were randomized to either continuation of HF diet ad libitum (OBESE group), caloric restriction with normal chow (Sniff Diets, Soest, Germany; R-NC group) or caloric restriction with HF diet (D12492, Research Diets Inc; R-HF group). The restriction consisted of individualized daily allowance so that the mice in both restriction groups achieved similar BW by the end of the 8 week intervention period as the age-matched control mice (LEAN) that had received ad libitum normal chow diet from the age of 8 weeks old onwards. Dietary macrocomposition is summarized in Table 1 and detailed composition is available in S1 Table. Based on previous experiments in our lab, we calculated that we would need 9–11 mice per experimental condition to show an expected difference in the area under the curve (AUC) of blood glucose levels of 10000 mg/dl*120min during the intraperitoneal glucose tolerance test (IPGTT) assuming a standard deviation (SD) of a 10000 mg/dl*120min and for 0.8 power and a type I error probability of 0.05. We anticipated failure to attain the body weight matching in the R-NC and R-HF groups (n = 15), but no mice were excluded from the final analyses. All animal experimental procedures were approved by the Ethics Committee of the University of Leuven (P085/2013) and the Guide for the care and use of laboratory animals, Eighth edition (2011) (http://grants.nih.gov/grants/olaw/guide-for-the-care-and-use-of-laboratory-animals.pdf) was followed.

**Table 1:** Macronutrient composition.

Label		Normal chow	High fat
Supplier, product ID		ssniff^®^ R/M-H	Research Diets D12492
Element	unit	per kg diet	per kg diet
ENERGY			
as fat	%	9.0	60.0
as carbohydrate	%	58.0	20.0
as protein	%	33.0	20.0
FAT			
total	g	32.5	254.5
saturated	g	5.7	81.5
MUFA	g	6.5	91.5
PUFA	g	20.3	81.5
CARBOHYDRATE			
total	g	412.0	314.6
complex	g	365.0	161.3
simple sugars	g	47.0	88.8
cellulose	g	NS	64.5
PROTEIN			
total	g	190.0	261.9

### Food intake and body composition

During the 8 week dietary intervention phase, all mice were single housed and weighed weekly. Food was supplied once per week for the ad libitum groups (LEAN, OBESE) and once daily for the restricted groups (R-NC and R-HF). Caloric intake was measured weekly for the ad libitum groups, by subtracting the weight of the remaining food pellets from the weight of total food supplied during that week. For the restricted groups, the caloric intake was calculated as the sum of daily allowance minus eventual left-overs from previous days. The mean daily caloric intake for the intervention period was calculated by dividing the total intake by the number of experimental days. Body composition of the total body with exclusion of the cranium was analyzed by dual-energy X-ray absorptiometry (DXA; PIXImus densitometer; Lunar, Madison, WI; software version 2.10.041) in anesthetized mice (phenobarbital 50 mg/kg, Sanofi Santé Animale, Brussels, Belgium).

### Indirect calorimetry

Mice were individually housed in automated cages for indirect calorimetry (TSE Phenomaster Calocages, Bad Homburg, Germany) in a room with 22°C ambient temperature and a 12 h-dark/light cycle, as described [15]. All mice had ad libitum access to water and food, except R-NC and R-HF mice, whoms food basket opening was automatically coupled to open upon actual food consumption in ad libitum fed mice in the adjacent cages. This prevented compensatory excess intake in restricted groups. Food intake, oxygen consumption, carbon dioxide production, and ambulatory activity were recorded over a 48 h period, but only the last 24 h were used for calculations, to exclude new cage environment bias. Respiratory exchange ratio (RER) and heat production were calculated as described [16].

### In vivo glucose homeostasis tests

Intraperitoneal glucose and insulin tolerance tests (IPGTT and ITT) were performed at 28 and 29 weeks of age, respectively. After 6 h fasting (8:00 to 14:00), mice were weighed and fasting glycaemia was determined on tail vein blood (Accu-Chek Aviva glucometer, Roche Diagnostics, Vilvoorde, Belgium). Glucose (2 g/kg BW) or insulin (0.75 mU insulin/g BW, Actrapid, NovoNordisk, Denmark) was administered, followed by measurement of glycaemia levels after 15, 30, 60, 90, and 120 min [17].

### Serum and tissue analyses

At 30 weeks of age, tail vein glycaemia was determined and the mice were sacrificed by carbon dioxide gassing, followed by blood collection by cardiac puncture and collection of epididymal white adipose tissue (eWAT) pads and liver. Serum was obtained by centrifugation for 10 min at 2000 g. Insulin levels were determined using a commercial ELISA kit (Mercodia, Huissen, Netherlands) on a Victor Multilabel Counter (Perkin Elmer, Wallac, Finland). Serum alanine minotransferase (ALT) levels were determined on a AU640 Biochemistry Analyzer (Beckman Coulter Inc., Brea, CA). Liver triglyceride (TG) levels were measured using the Triglyceride Quantification Kit colorimetric assay (Abcam, Cambridge, UK).

### Histology and immunohistochemistry

Samples of liver and eWAT were fixed in paraformaldehyde 4%, and embedded in paraffin. Serial 5 μm thick sections were stained with hematoxylin and eosin (H&E) and liver sections were stained with Sirius Red for hepatic fibrosis. Photomicrographs (magnification 20x for adipose, 10x for liver tissue) were taken on a Zeiss Axiovert microscope using Axiovision software (Zeiss, Zaventem, Belgium).

Adipocyte cell size was determined in 3 different microscopic fields per fat pad by counting at least 100 cells using ImageJ software (NIH, Bethesda, MD) with commands “analyze-set scale”, “process–substract background”, “image-threshold”, “process-make binary” and “Measure and Label Macro”, as described [18].

All liver samples were analyzed by an expert liver pathologist, blinded for dietary condition or surgical intervention. Steatosis, activity and fibrosis were semi-quantitatively scored according to the NASH-Clinical Research Network criteria [19]. The amount of steatosis (percentage of hepatocytes containing fat droplets) was scored as 0 (<5%), 1 (5–33%), 2 (>33–66%) and 3 (>66%). Hepatocyte ballooning was classified as 0 (none), 1 (few) or 2 (many cells/prominent ballooning). Foci of lobular inflammation were scored as 0 (no foci), 1 (<2 foci per 200× field), 2 (2–4 foci per 200× field) and 3 (>4 foci per 200× field). Fibrosis was scored as stage F0 (no fibrosis), stage F1a (mild, zone 3, perisinusoidal fibrosis), stage F1b (moderate, zone 3, perisinusoidal fibrosis), stage F1c (portal/periportal fibrosis), stage F2 (perisinusoidal and portal/periportal fibrosis), stage F3 (bridging fibrosis) and stage F4 (cirrhosis).

### Quantitative polymerase chain reaction

Total RNA of eWAT and liver tissue was extracted from 50 mg tissue using the RNeasy Lipid Tissue Mini Kit (Qiagen, Hilden, Germany) or SV Total RNA Isolation System (Promega, Madison, WI) respectively. First-strand cDNA was synthesized using Superscript II RT (Life Technologies, Ghent, Belgium). qRT-PCR was performed using a StepOne real-time PCR system (Applied Biosystems, Ghent, Belgium) and Fast SYBR Green Mastermix (Qiagen) or TaqMan mastermix (Life technologies), based on the ΔΔCt quantification method. Values were normalized to the geometric mean of housekeeping genes 60S ribosomal protein L27 and hypoxanthine-guanine phosphoribosyltransferase. Primer sequences are listed in S2 Table.

### Statistics

Data are presented as mean ± SEM, unless otherwise indicated. Statistical analyses were performed with GraphPadPrism 6.0 (GraphPad Software, San Diego, CA). Data were compared by one way Analysis of variance (ANOVA) followed by post hoc analysis with Bonferroni multiple-comparison test, unless otherwise stated in figure legends. A Pearson correlation test was performed to explore associations. Differences were considered statistically significant at P < 0.05.

## Results

### Body weight, caloric intake and body composition

**Fig 1:**
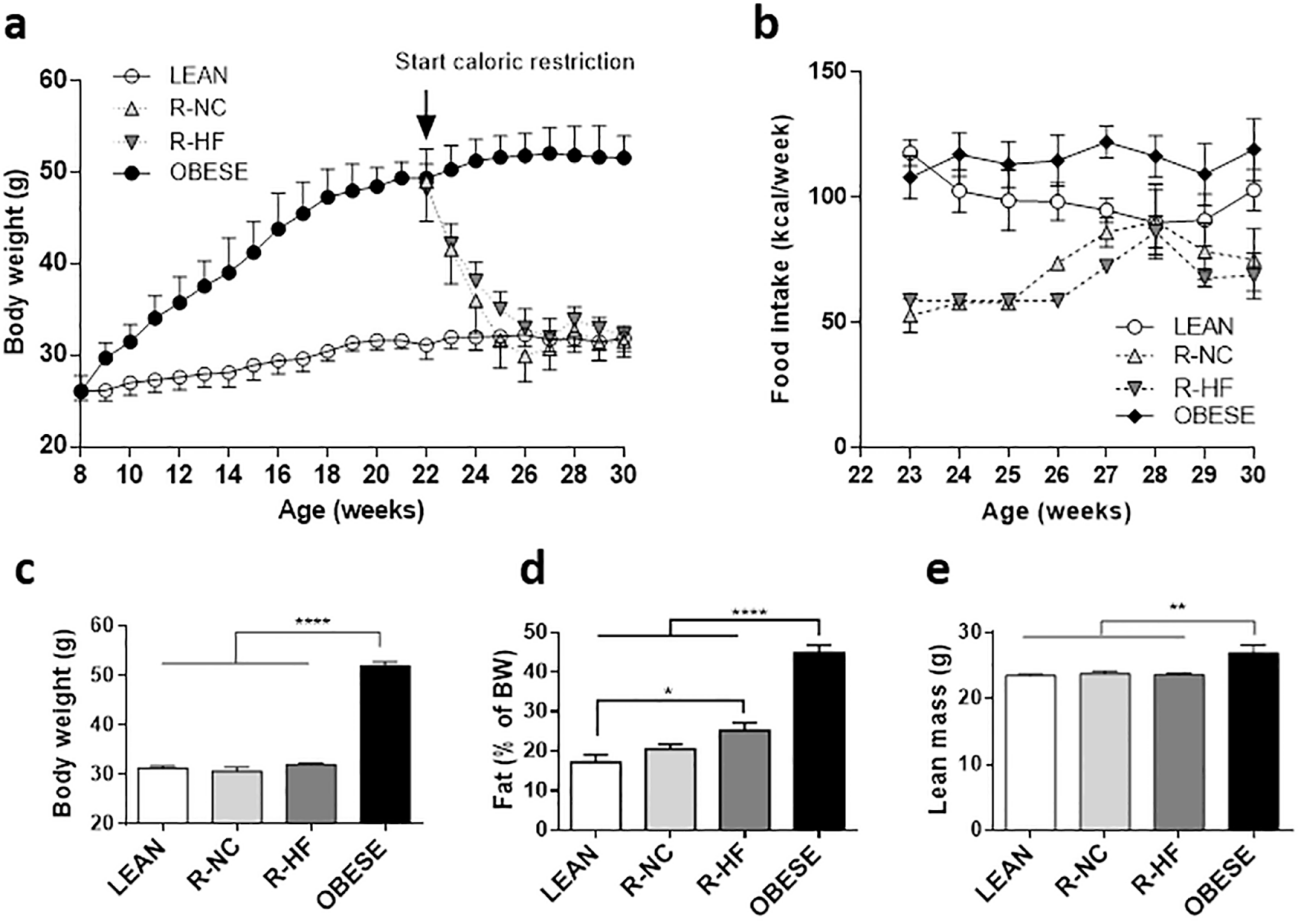
Body weight and body composition. Body weight evolution (a) and caloric intake (b) LEAN (white circles), R-NC (light grey upward triangles), R-HF (dark grey downward triangles) and OBESE (black circles) mice. N = 11 for LEAN and OBESE, n = 15 for R-NC and R-HF. Body weight (c) and body composition, expressed as fat percentage (d) and lean mass (e) as measured by DXA at 30 weeks of age (n = 6 per group). Data are presented as mean±SEM * P<0.05; ** P<0.01 *** P<0.001 **** P<0.0001.

BW evolution is shown in Fig 1a. At the start of the dietary intervention (22 weeks of age), the mice on a NC diet weighed 31.1 ± 1.5 g while the HF diet fed mice weighed 48.5 ± 3.5 g. The total caloric intake during the 8 week intervention period was 634 ± 97 kcal for OBESE mice, 536 ± 84 kcal for LEAN mice, 428 ± 51 kcal for R-NC fed mice and 391 ± 49 kcal for R-HF mice (no significant difference; Fig 1b). At 30 weeks of age, OBESE mice weighed 162% of LEAN mice (51.7 ± 2.4 g vs 31.9 ± 1.4 g (P < 0.001), while the BW of R-NC (31.6 ± 1.7 g) and R-HF (32.3 ± 0.9 g) mice was similar to that of LEAN mice (31.9 ± 1.4 g), as planned in the experimental design (Fig 1c). DXA showed a high body fat percentage in OBESE mice (44.9 ± 2.0% of BW) compared to all other groups (P < 0.0001 vs LEAN/R-NC/R-HF). While LEAN and R-NC fed mice had a similar fat percentage (resp. 17.2 ± 1.9% and 20.6 ± 1.2%), R-HF mice had more fat (25.3 ± 1.9%, P 0.0267) (Fig 1d). Lean mass of OBESE mice was slightly higher (113%) than in LEAN mice and mice that lost weight (P 0.029 vs. LEAN/R-NC/R-HF) (Fig 1e).

### Energy expenditure

**Fig 2:**
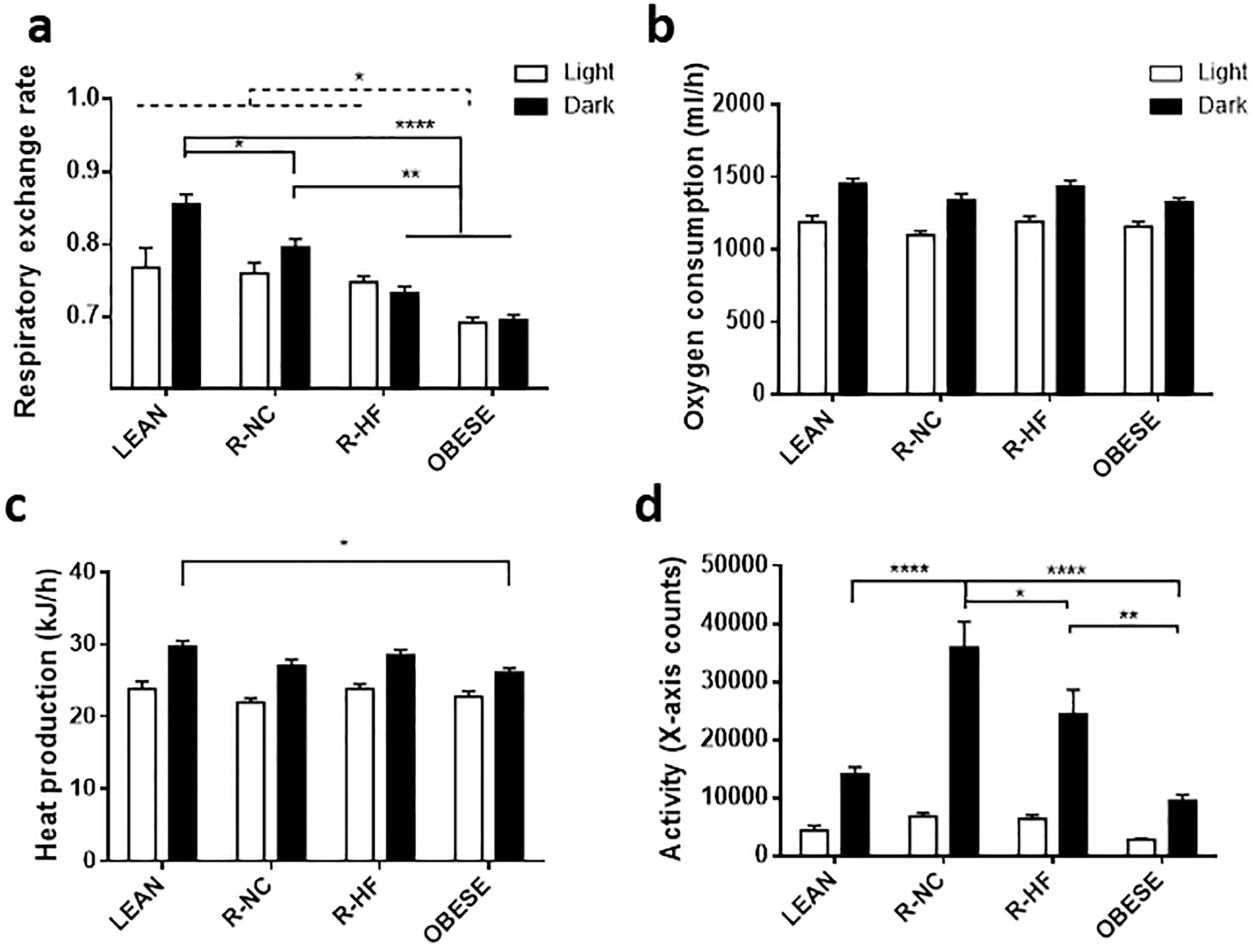
Energy expentidure components as obtained in automated cages for indirect calorimetry. Respiratory exchange rate (a), oxygen consumption (b), heat production (c) and ambulatory activity (d) (n = 6–8 per group). Data are presented as mean±SEM * P<0.05; ** P<0.01 *** P<0.001 **** P<0.0001.

The respiratory exchange rate (RER) of OBESE mice was 0.69 ± 0.01 during the light phase and 0.70 ± 0.01 during the dark phase, indicative of fat oxidation as main energy substrate. During the light phase, which is the inactive phase for mice, the LEAN and calorie restricted mice had a slightly higher RER than the OBESE mice (0.76 ± 0.03 for LEAN, 0.76 ± 0.03 for R-NC and 0.75 ± 0.04 for R-HF, all P < 0.05 vs. OBESE). During the active, dark phase, the RER was higher when carbohydrate was an available energy source: in the normal chow groups (LEAN 0.85 ± 0.01 and R-NC 0.80 ± 0.02) compared to the HF groups (R-HF 0.73 ± 0.01 and OBESE 0.70 ± 0.01). The RER of R-NC mice during the dark phase was however lower than the RER of LEAN mice (P 0.0221) (Fig 2a). There were no differences in oxygen consumption, nor was there a significant difference in total energy expenditure between R-NC and R-HF mice. There were also no differences in ambulatory activity between LEAN (14079 ± 1235 counts) and OBESE (9561 ± 987 counts) mice, but both R-NC (36021 ± 4430 counts) and R-HF (24505 ± 4140 counts) mice were more active during the dark phase than OBESE mice (P < 0.01). Moreover, R-NC mice were more active than R-HF mice during the dark phase (P 0.0022).

### Effects on adipose tissue

**Fig 3:**
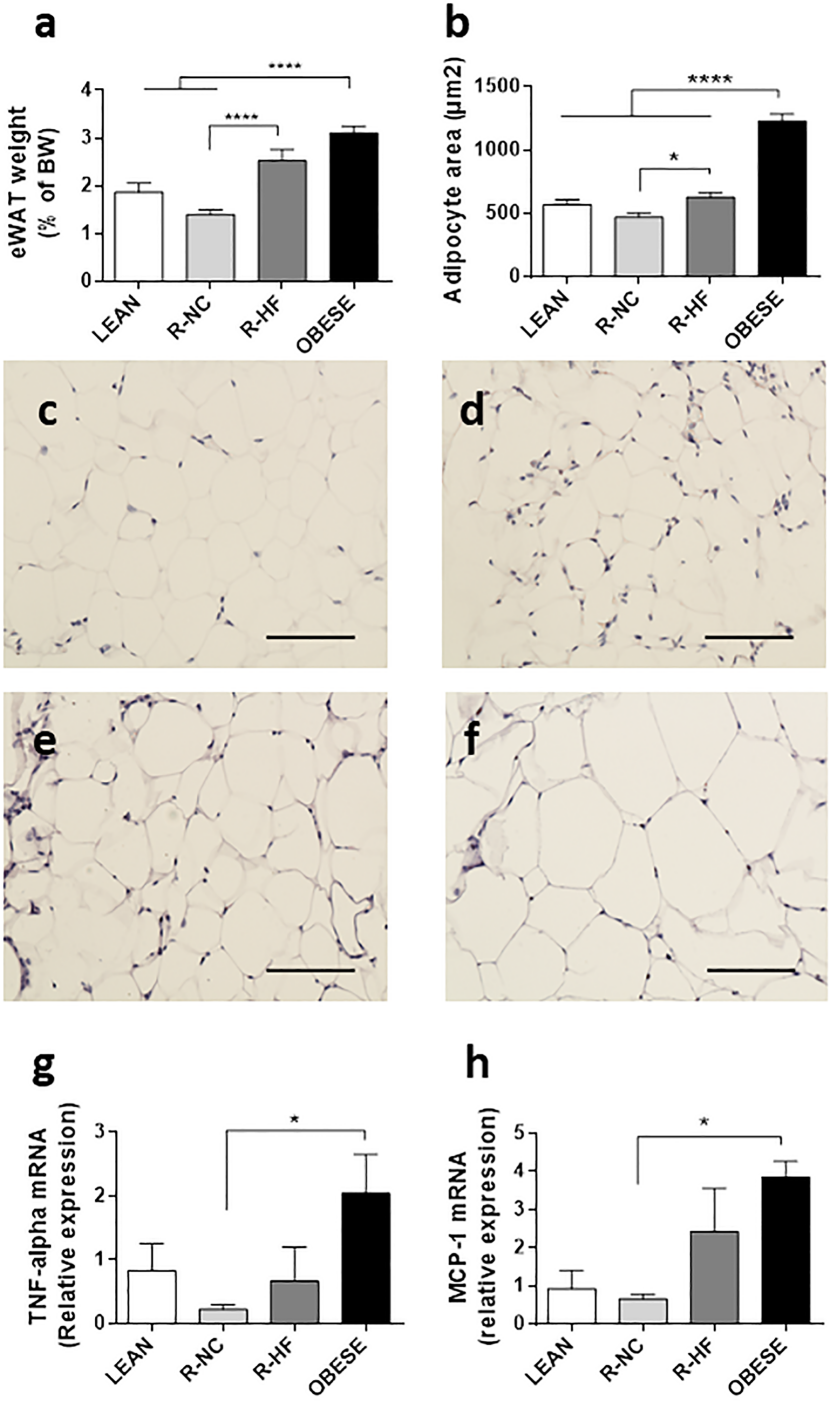
Characteristics of epididymal white adipose tissue (eWAT) upon different weight loss interventions. Relative weight of eWAT pads (n = 11–15 per group) (a) Adipocyte size (b) as based on microscopic H&E stained sections of eWAT of LEAN (c), R-NC (d), R-HF (e) and OBESE (f) mice (magnification 20x, scale bars indicate 100μm). Average adipocyte surface in eWAT tissue samples (n = 6–9 per group, G) and relative mRNA expression levels of TNF-alpha (g) and MCP-1 (h) in eWAT (n = 6–8, per group). Data are presented as mean±SEM. * P<0.05; *** P<0.001.

The relative size of the eWAT pads in OBESE mice (3.1 ± 0.1% of BW) was 1.6 times larger than in LEAN mice (1.9 ± 0.2% of BW) and 2.2 times larger than in R-NC mice (1.4 ± 0.1% of BW) (both P < 0.001 vs OBESE) (Fig 3a). The eWAT pads of R-HF mice (2.6 ± 0.2% of BW) were similar in size as those of OBESE (3.1 ± 0.1% of BW) mice, and R-NC pads were only 54% the size of those of R-NC mice (1.4 ± 0.1% of BW, P < 0.0001; Fig 3a). The average adipocyte surface in the eWAT pads of OBESE mice was about 2-fold larger than in LEAN mice (1226 ± 59 vs 567 ± 39 μm^2^, P < 0.0001) and both weight loss groups (468 ± 34 μm^2^ in R-NC mice, 625 ± 39 μm^2^ in R-HF mice; all P < 0.0001 vs OBESE). The adipocytes of R-HF mice were 134% of those in R-NC fed mice (P 0.0285 vs R-HF) (Fig 3b). We could not detect differences in mRNA expression of markers of lipogenesis/lipolysis for beta-oxidation that could explain the differences between R-NC and R-HF at this time point (Figures A-C in S1 Fig).

However, the mRNA level of the inflammatory cytokine tumor necrosis factor alpha (TNF-α) and the Monocyte Chemoattractant Protein-1 (MCP-1) were reduced in the eWAT pads of R-NC mice compared to the fat pads of OBESE mice (P 0.042 for for TNF-α and P 0.0318 for MCP-1 for OBESE vs. R-NC), these genes were not downregulated in eWAT of R-HF mice, compared to OBESE mice (Fig 3g and 3h).

### Effect on liver tissue

**Fig 4:**
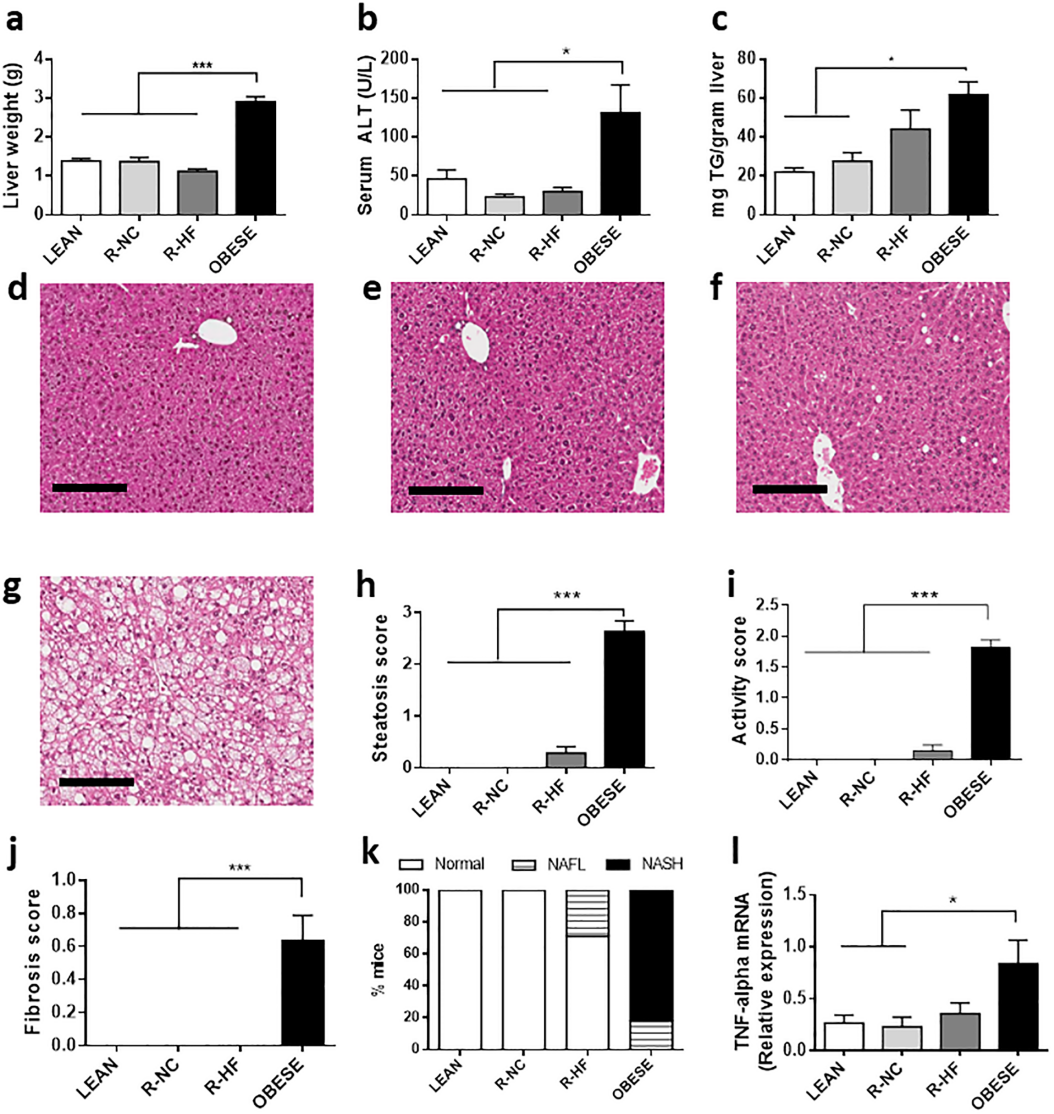
Characteristics of liver tissue upon different weight loss interventions. Relative liver weight (n = 11–15 per group) (a) Serum alanine aminotransferase (ALT) levels (n = 6–9 per group) (b) and hepatic triglyceride (TG) content (n = 6 per group) (c). H&E stained sections of liver tissue from LEAN (d), R-NC (e), R-HF (f) and OBESE (g) mice (magnification 10x, scale bars indicate 200μm). Steatosis (h), Inflammatory activity (i) and Fibrosis (j) score and Relative frequency of liver disease (n = 11–15 per group) (k); Relative mRNA expression levels of TNF-alpha (n = 6–8 per group) (l). Data are presented as mean±SEM. * P<0.05; *** P<0.001.

The liver mass was 2-fold higher in OBESE (2.9 ± 0.1 g), compared to LEAN (1.4 ± 0.1 g), R-NC (1.4 ± 0.1 g) and R-HF (1.1 ± 0.01) mice (P 0.3293 for R-NC vs R-HF; Fig 4a). Alanine aminotransferase (ALT) levels were increased in OBESE vs LEAN and calorie restricted mice (P < 0.05 for OBESE vs. LEAN/R-NC/R-HF) (Fig 4b). The hepatic TG content was almost 3 times higher in OBESE than in LEAN mice (61.9 ± 6.7 vs 22.1 ± 2.1 mg TG/g liver, P 0.0024) (Fig 4c). Whereas the hepatic TG content in R-NC mice (27.6 ± 4.3 mg TG/g liver) was also lower than in OBESE mice (P 0.0092), the TG content in livers of R-HF mice was in between that of the LEAN and OBESE mice, but the difference did not reach statistical significance (P 0.4219 vs OBESE and P 0.1328 vs LEAN) (Fig 4c). We could not detect any differences in fatty acid translocase CD36 mRNA expression in the liver between R-NC and R-HF groups at tihis time point that could explain the differences in steatosis (S2 Fig). However, histological examination confirmed the absence of hepatic steatosis in LEAN mice, whereas livers from all OBESE mice showed steatosis, affecting the majority of the hepatocytes (stage 3), resulting in a steatosis score of 2.6 ± 0.7 out of 3 (P < 0.001 for OBESE vs. all other groups) (Fig 4d–4h). None of the R-NC mice’s samples showed steatosis, whereas fat accumulation was apparent in 4/14 (28.6%) of the R-HF mice’s liver samples (all stage 1), resulting in a steatosis score of 0.29 ± 0.13 for R-HF mice (not significant P 0.5025 vs. LEAN and P 0.3951 vs R-NC) (Fig 4h). In the liver samples of OBESE mice, prominent ballooning was seen in 9/11 (81.8%) of the cases (P < 0.001 vs. all groups), and 8/11 (72.7%) of the livers showed signs of fibrosis (P < 0.001 vs. all groups), in contrast to all other groups, that were free of fibrotic tissue (P < 0.001 vs. all groups, Fig 4j). Livers from LEAN and R-NC mice showed no inflammatory activity, while in 2/14 (14.2%) in R-HF mice some inflammation was seen (not significantly different from LEAN or R-NC) (Fig 4i). Overall, OBESE mice tended to have NASH, while some R-HF mice had signs of NAFLD and all LEAN and R-NC looked normal. (Fig 4k)

Indeed, TNF-α transcript levels in liver tissue of OBESE mice showed a 3.1 fold induction compared to liver tissue from LEAN mice (P 0.0435 vs OBESE). In livers from R-NC mice, TNF-α expression was similar and significantly lower than in OBESE livers (P 0.0131 vs. OBESE) (Fig 4j). In R-HF mice however, the mRNA level of TNF-α was not significantly different from OBESE mice (P 0.0855) (Fig 4l).

### Effects on glucose homeostasis

**Fig 5:**
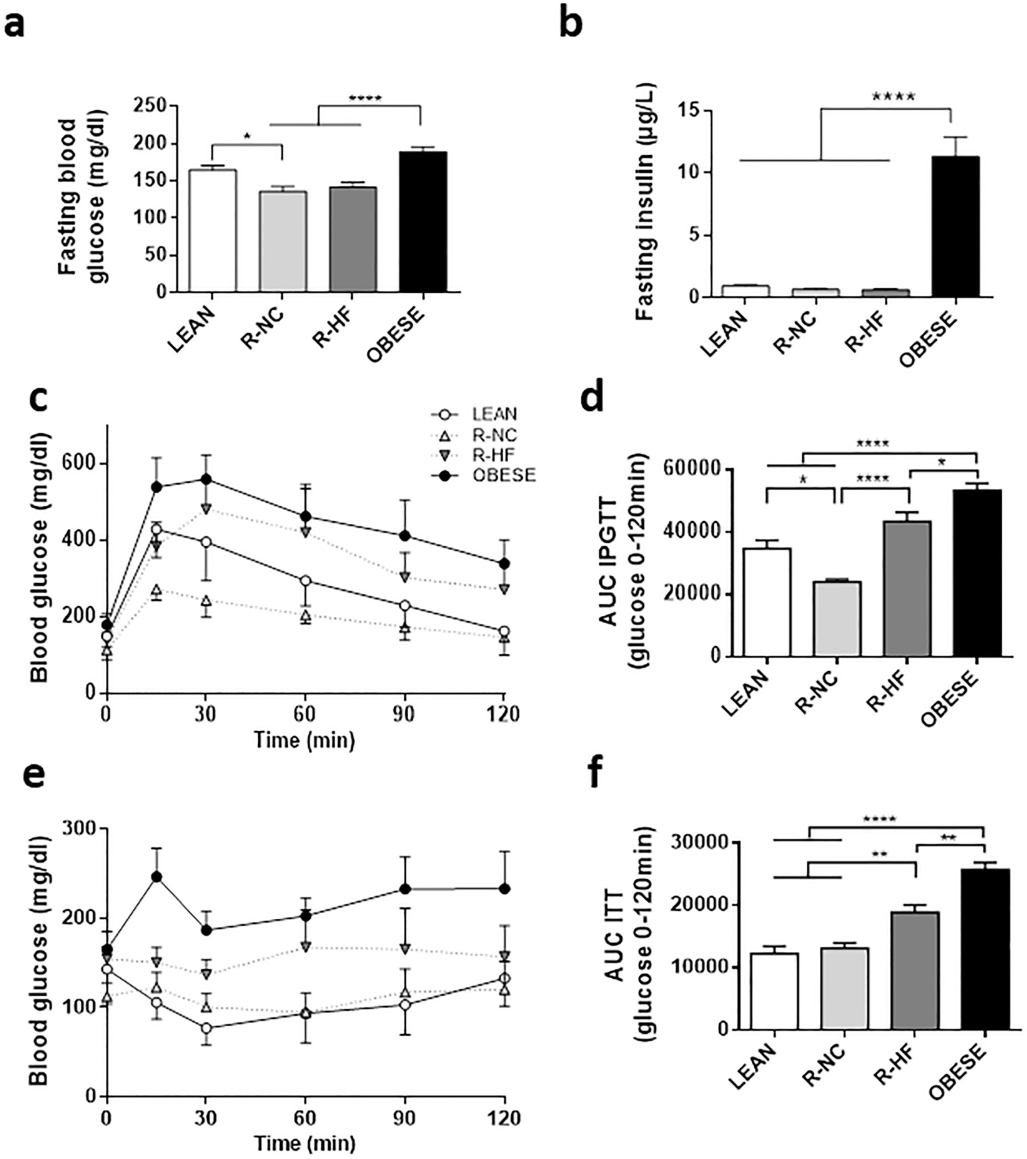
Glucose homeostasis parametes upon weight loss interventions. Blood glucose (n = 11–15 per group) (a) and serum insulin levels (n = 6 per group) (b) after 6h fasting. Intraperitoneal glucose tolerance test, and calculated AUC (n = 8 per group) (c, d) Insulin tolerance test and calculated AUC (n = 6 per group). Data are presented as mean±SEM. * P < 0.05; *** P < 0.001 **** P < 0.0001.

Fasting blood glucose level tended to be higher in OBESE (189 ± 10 mg/dl) than in LEAN controls (164 ± 6 mg/dl, P 0.0849). However, the fasting glycaemia of R-NC (136±6 mg/dl) and R-HF (141±7 mg/dl) mice was lower than that of OBESE mice (P < 0.001 for R-NC/R-HF vs. OBESE) (Fig 5a). OBESE mice were hyperinsulinemic (11.3 ± 3.9 μg/l) compared to all other groups (all P < 0.001, Fig 5b).

The OBESE mice were very glucose intolerant, compared to all other groups, as evidenced by a very high area under the curve (AUC) of blood glucose levels during the IPGTT (53348 ± 2214 mg/dl*120min, P < 0.05 compared to all other groups, Fig 5c and 5d). R-NC mice had the best glucose tolerance, as evidenced by a 69% of AUC IPGTT of that in LEAN mice (24022 ± 839 vs 34635 ± 2670 mg/dl*120min, P 0.0168 vs. LEAN) (Fig 5d). For R-HF fed mice (43384 ± 2872 mg/dl*120min) on the other hand, the AUC IPGTT tended to be higher than LEAN control mice (125%, P 0.0696 vs LEAN) (Fig 5d).

Upon ITT, the highest AUC was measured in OBESE controls (25698 ± 1138, mg/dl*120min, P < 0.0001 vs. all other groups, Fig 5e and 5f). The R-HF mice had a higher insulin resistance than LEAN controls (18823 ± 1202 vs 12266 ± 1142 mg/dl*120min, P 0.0024), but also compared to R-NC mice (13108 ± 848 mg/dl*120min, P 0.0084 < 0.05) (Fig 5f).

### Correlations between metabolic parameters

To evaluate the best indicators of glucose intolerance and NAFLD, we examined the correlations between metabolic parameters (Table 2). At thirty weeks of age, the total BW correlated well with size of the eWAT fat pads (r = 0.43, P = 0.018), hepatic TG content (r = 0.59, P < 0.001) and histological signs of hepatic steatosis (r = 0.87, P < 0.001). Moreover, BW was strongly correlated with insulin resistance as expressed by fasting insulin levels (r = 0.87, P < 0.001), HOMA-IR (r = 0.88, P < 0.001) and ITT AUC (r = 0.77, P < 0.001). A direct relation between BW and fasting glycaemia (r = 0.57, P < 0.001) and glucose tolerance (IPGTT AUC, r = 0.57, P < 0.001) was also observed.

**Table 2:** Correlations between body composition, glucose parameters, epididymal white adipose tissue (eWAT) size, hepatic steatosis and ALT levels.

	**BW**	**FPG**	**Insulin**	**IPGTT AUC**	**ITT AUC**	**eWAT %BW**	**TG content**	**Steatosis**	**ALT**
**BW**		0.57 ***	0.87 ***	0.57 ***	0.77 ***	0.43 *	0.59 ***	0.87 ***	0.46
**FPG**	0.57 ***		0.58 ***	0.32	0.55 ***	0.25	0.29	0.62 ***	0.42
**Insulin**	0.87 *	0.58 ***		0.58 ***	0.77 ***	0.40 *	0.52 **	0.75 ***	0.62 **
**IPGTT AUC**	0.57 *	0.32	0.58 ***		0.67 ***	0.77 ***	0.49 **	0.61 ***	0.58 *
**ITT AUC**	0.77 *	0.55 **	0.77 ***	0.67 ***		0.50 **	0.60 ***	0.81 ***	0.74 ***
**eWAT %BW**	0.43 *	0.25	0.40 *	0.77 ***	0.50 **		0.61 ***	0.53 **	0.51 *
**TG content**	0.59 *	0.29	0.52 **	0.49 **	0.60 ***	0.61 ***		0.56 ***	0.56 *
**Steatosis**	0.87 *	0.62 ***	0.75 ***	0.61 ***	0.81 ***	0.53 **	0.56 ***		0.61 **
**ALT**	0.46	0.42	0.62 **	0.58 *	0.74 ***	0.51 *	0.56 **	0.61 **	

Correlations between body composition, glucose parameters, epididymal white adipose tissue (eWAT) size, hepatic steatosis and ALT levels. BW body weight; FPG Fasting plasma glucose; IPGTT intraperitoneal glucose tolerance test; ITT insulin tolerance test; TG triglyceride; ALT Alanine Aminotransferase. Values are R correlation coefficient,

* denotes P<0.05

** P<0.01

*** P<0.001.

IPGTT AUC correlated with the relative eWAT size (r = 0.76, P < 0.001) and to a lesser extent with the hepatic TG content (r = 0.61, P < 0.001). ITT AUC on the other hand, correlated more with hepatic steatosis (r = 0.81, P < 0.001) and serum ALT (r = 0.74, P < 0.001) than eWAT size (r = 0.50, P < 0.01).

The size of the eWAT fat pads correlated well with hepatic TG content (r = 0.61, P < 0.001) and histological signs of fatty liver (r = 0.53, P < 0.01). In turn, hepatic steatosis and TG content both correlated with serum ALT levels (r = 0.61 and P < 0.01 for steatosis; r = 0.56 and P < 0.01 for TG content).

## Discussion

Calorie restriction on normal chowand high fat diet resulted in identical weight loss, but mice that lost weight on a NC diet had less intra-abdominal fat and better glucose tolerance than mice that were dieting on high fat.

While most caloric restriction studies have focussed on the amount of weight lost [20], here we show the importance of the dietary composition. Our data suggest that a high fat diet should be avoided and a balanced, low fat diet should be used in order to maximize metabolic benefit. Indeed, despite similar weight loss and similar BW at the end of the study, mice that underwent caloric restriction on a HF diet, had a slightly higher fat percentage and substantially larger epidydymal fat pads than R-NC mice. This suggests that the continued oversupply of saturated fat in the R-HF group is still preferentially stored in visceral, metabolically unhealthy sites, and not in subcutaneous fat. Earlier studies in humans showed less fat loss when consuming a HF diet during weight loss [6]. Presumably, persistent fat oxidation in low fat diet intervention groups and the resulting increased fat loss, underlies the superior metabolic benefit observed with low fat diets [7]. In our study, the RER during the dark phase was slightly higher in R-NC mice than in R-HF mice, indicating that R-NC not only use fat as an energy substrate but also some carbohydrate, as provided in the NC diet. However, the RER in NC-R mice was lower than in LEAN mice that consumed a NC diet, confirming persistent fat oxidation in R-NC mice.

Our results do not only provide definitive proof of a substantially better metabolic effect of low fat normal chow versus HF caloric restriction, it also elucidates the pathophysiological mechanisms that underly this superior effect: i.e. larger eWAT fat pads and larger adipocyte size in the R-HF group. Noteworthy is that the adipocytes in R-NC fed mice were not enlarged compared to LEAN control mice, but appeared to have a higher cellular density. This suggests that adipocyte’s size rather than number is affected by weight loss. As enlargement of adipocytes is thought to be the main driver for obesity related low grade inflammation and insulin resistance [21], the enlarged adipocytes in R-HF fed mice provide a mechanistic link between the dietary composition and the observed different effect on glucose tolerance. Indeed, mRNA levels of the inflammatory cytokine TNF-α and the Monocyte chemotactic protein 1 (MCP-1) were significantly lower in the eWAT of R-NC fed, but not in R-HF fed mice compared to OBESE mice, suggesting persistent inflammation in the eWAT of R-HF fed mice. As in adipose tissue, the mRNA expression of TNF-α was normal in liver tissue of R-NC, but not in R-HF mice, confirming the association of adipose tissue inflammation and NAFLD [22].

The thorough evaluation of glucose homeostasis is a major asset of the current study, since most head-to-head dietary composition studies have only focused on the magnitude of weight loss [5,23]. In our study, fasting blood glucose and fasting insulin levels improved in both weight loss groups (R-NC and R-HF) compared to OBESE mice, suggesting a similar improvement of hepatic glucose output. However, although glucose disposal in R-NC mice was at least as good as in LEAN mice, both the IPGTT and the ITT showed (residual) glucose intolerance in R-HF mice, despite similar weight loss. Noteworthy is that the improved glucose tolerance in the restriced mice is to a large extent weight loss dependent, though the difference between the R-NC and R-HF in metabolic improvement clearly demonstrates that there is also an influence of the dietary composition at similar weight loss. Glucose and insulin tolerance tests were tightly correlated with eWAT size and hepatic TG content.

This study has a number limitations. First, it remains unclear to which extent these murine data can be translated to humans. However, the rodent model allowed us to loose all excessive body weight while stringently controlling the diet, which would not be possible in human subjects [4,24]. Second, we opted to use two commercially available and widely used diets, namely a “standard” diet, i.e. normal chow diet which considered healthy and adequate for rodents, and compare it to a widely used “extremely unhealthy diet” [25,26]. We acknowledge that this results in many differences in quantity and quality of fat, carbohydrate and protein between the used diets. However, in our view, studies on the exact and ideal quantity and quality of fat, carbohydrate and protein should be performed in humans ideally. We provide the general proof of principle that changing the macronutrient composition can powerfully influence the metabolic outcome despite similar weight loss. The contribution of the proportion and the nature of the dietary macronutrient composition needs further clarification in humans, and are beyond the scope of the current study which was to evaluate whether metabolic improvement differs at identical weight loss with a different diet composition. Although both the amount of weight loss and the difference in dietary composition in this study are drastic, the changes in body composition and energy expenditure are fully in line with previous reports in human studies [7,27]. Thus, we are confident that our findings are relevant for the human situation. In this respect, we have also refrained from using a forced overfeeding protocol in this study to assess whether there would also be weight independent differences on metabolic parameters depending on the dietary composition when a positive energy balance is maintained. Such overfeeding protocols are invasive, due to the need for continuous intragastric feeding with a liquid diet [28] and our main aim was to assess the metabolic effects of weight loss. However, more research is warranted to assess whether the same differential weight independent metabolic effects are observed when a positive energy balance is maintained using diets of varying macronutrient compositions.

Finally, further studies are needed to elaborate the underlying mechanisms and pathways that facilitate metabolic improvement on different dietary macronutrient situations. This would require more detailed analysis during weight loss in stead of the weight stable phase only as is the case in the current study, but also other tissues including subcutaneous fat, brown fat, muscle, hypothalamus and gut micribiome are most likely also affected by drastic dietary interventions.

In conclusion, caloric restriction can completely reverse the obesity-induced visceral fat infiltration and inflammation, if performed on a balanced, low fat diet. If similar caloric restriction is performed on a diet that is rich in saturated fats, residual adipocyte hypertrophy, hepatic steatosis and low grade inflammation attenuate the metabolic benefits of weight loss. Therefore, low fat diets should be preferred to high fat diets.

## Supporting information

S1 Fig mRNA expression profile peroxisome proliferator activated receptor (PPARy) or sterol regulatory element binding transcription factor 1 (SREBF1), both markers of lipogenesis, and uncoupling protein 2, a marker of beta-oxidation, in eWAT (a, b, c). (DOCX)

S2 Fig mRNA expression of fatty acid translocase CD36 expression in liver tissue. (DOCX)

S1 Table Detailed dietary composition. (XLSX)

S2 Table Primers used for RT-qPCR. FW = Forward, RV = Reverse, TP = Taq Probe.
(XLSX)

## Abbreviations

ALT: alanine aminotransferase
AUC: area under the curve
BW: body weight
DXA: dual-energy X-ray absorptiometry
eWAT: epididymal white adipose
HF: high fat
IPGTT: intraperitoneal glucose tolerance test
ITT: insulin tolerance test
MCP-1: monocyte chemoattractant protein-1
NAFLD: non alcoholic fatty liver disease
RER: respiratory exchange ratio
R-HF: restriction high fat diet
R-NC: restriction on normal chow
TG: triglyceride
TNF-α: tumor necrosis factor alpha
