# Supplementary material for: At similar weight loss, dietary composition determines the degree of glycemic improvement in diet-induced obese C57BL/6 mice
Source: PLoS One. 2018 Jul 23;13(7):e0200779. doi: 10.1371/journal.pone.0200779 (PMC6056053; doi:10.1371/journal.pone.0200779)
Supplement: S1 Fig — (DOCX) [file pone.0200779.s001.docx]

Supplemental figure 1. mRNA expression of peroxisome proliferator activated receptor (PPARy) or sterol regulatory element binding transcription factor 1 (SREBF1), both markers of lipogenesis, and uncoupling protein 2, a marker of beta-oxidation, in eWAT (a, b, c).

**a b c**
