# Supplementary material for: At similar weight loss, dietary composition determines the degree of glycemic improvement in diet-induced obese C57BL/6 mice
Source: PLoS One. 2018 Jul 23;13(7):e0200779. doi: 10.1371/journal.pone.0200779 (PMC6056053; doi:10.1371/journal.pone.0200779)
Supplement: S2 Fig — (DOCX) [file pone.0200779.s002.docx]

Supplemental figure 2. mRNA expression of fatty acid translocase CD36 expression in liver tissue.
